# Just say “no”: Can dentists refuse care on the basis of finances? A survey using an ethical vignette in an Iranian Dental School

**DOI:** 10.1186/s12910-020-00554-7

**Published:** 2020-10-31

**Authors:** Ali Kazemian, Mahsa Fayyazi, Shahrzad Shafiee

**Affiliations:** 1 Department of Community Oral Health, School of Dentistry, Mashhad University of Medical Sciences, Vakilabad Blvd, P.O. Box 984, Mashhad, Iran; 2 School of Dentistry, Mashhad University of Medical Sciences, Mashhad, Iran

**Keywords:** Ethics, Dental Education, Financial Management, Questionnaire

## Abstract

**Background:**

Decision making when patients ask a dentist for fee reduction is a real ethical dilemma at dental settings. The aim of this study was to evaluate how dental students and tutors think about their position for, or against fee reduction at dental offices.

**Method:**

It was a questionnaire-based survey, which examined the ethical attitudes of students and tutors of an Iranian Dental School. The questionnaire included a vignette about an ethical dilemma at a dental office. Different ethical approaches, i.e. duty-based, virtue-oriented and consequentialist arguments, for or against fee reduction at dental office were suggested. Respondents were asked to rank those ethical options. Data was entered and analyzed in SPSS 16.0.

**Result:**

121 dental students and thirty-six faculty members (dental specialists) participated in this study. It revealed that a majority of dental students and tutors (68%) are in favor of charging patients less, when facing an imagined request at dental office, using either virtue-oriented (54%) or consequentialist (14%) argument for fee reduction. The difference between rankings of four options was statistically significant, while no statistically significant difference exists neither between male and female respondents, nor students and tutors.

**Conclusion:**

This case study provides a basis for fruitful discussions in ethics courses for dental students. Our study suggests that financial issues should be considered as a part of ethical training within the dental student's curriculum.

## Introduction

For both patients who need dental care, and for the dentists who treat them, financial issues are among the main questions that arise for both parties. Several surveys have shown that the high cost of treatment, and the financial limitations faced by many patients are the main barriers to accessing dental care [1–3]. These barriers inevitably have negative effects on oral health outcomes [4, 5]^.^ Policies that promote opportunities for patients with lower social-economic status to see a dentist may reduce the single most important impediment to regular dental care [6].

Furthermore, the financial discussions that must take place, affect the dentist-patient relationship, and are a routine challenge at dental settings. Offices that have an efficient, thoughtful, and context specific process for talking to their patients about the cost of treatment and the method of payment have higher financial profits, for having a straightforward initial discussion relieves uncertainty for both dentists and patients [7, 8]. Many of the treatment plans are rejected simply because the patient cannot afford to pay either at the time, or even over a period of weeks [9]. Because each dentist must confront the choice to offer fee reduction or refuse care where the request is made, we argue that it is a significant ethical and practical challenge as each decides, for example, how to react to the request from disadvantaged patients for reduction in their treatment fees. This situation is more likely to happen in oral health systems where out-of-pocket payment is dominant, but also is encountered when eligibility for exemptions or reduced prices, known as disease-specific responses, are adjudicated. Financial inaccessibility and inability to pay are typical system constraints [10].

“On average, do graduates benefitting from state tuition subsidies charge their patients less?” [11] This is one of the questions Bartolami raised in his reflection on dental graduates’ sense of gratitude for the public’s generosity. While charging patients less could be seen as a moral obligation of dentists, especially those who benefitted from public funds, or who have a practice with a significant economic advantages and generally well-paying patients, this obligation might be understood in other, but equally ethical ways.

Dentists tend to state that they choose their profession based on a desire to behave altruistically [12], which seems to be supported by prosocial attitude of dental students [11]. Focusing on professional issues, such as social and economic situations, which are related to patients’ non-medical problems, could be an effective way for discussing ethical issues of dental practice and promoting ethical sensitivity of dental students within the teaching of professional conduct. In this study, we studied how dental students and tutors thought about their position for, or against fee reduction at dental offices and the way they put forward their arguments to justify their position.

## Method

This study was a questionnaire-based survey, which examined the ethical attitudes of students and tutors of Mashhad Dental School, in Mashhad, Iran. The questionnaire was distributed in the Medical Ethics course to two sequential groups of six-year dental students in July and December 2017. Additionally, it was sent via email to the tutors of the School of Dentistry, Mashhad University of Medical Sciences, in Mashhad, Iran. Those who did not respond in a week received a reminder. While all the students at the classes completed the questionnaire, the response rate of the tutors to our online survey was 40.0%. The study was approved by the Institutional Review Board of Mashhad Dental School.

The questionnaire included a vignette about an ethical dilemma at a dental office (Additional file 1). The vignette describes a meeting of five dentists. The host, Dr. E. raises an issue regarding his difficulty in decision making when patients ask him or her for fee reduction. Dr. E. wants to know how his or her colleagues behave in such circumstances, and how they think about this problem.

Four approaches are then taken by the colleagues of Dr. E. They include:

A Consequentialist argument *against* fee reduction—Dr. A. believes if a dentist began to accept requests for fee reduction, it would gradually raise other patient’s expectations, which may result in unfavorable consequences.
B Virtue-oriented argument *for* fee reduction—Dr. B. considers maintaining the virtuous character of the dentist as the most important response to the dilemma. A good dentist could not remain indifferent about the unaffordable tariffs for treatments for his or her patient, and could never deny care.
C Duty-based argument *against* fee reduction—According to Dr. C. making fee reduction is never listed as a dentist's duty. We have to be careful to adhere to our obligations, not necessarily more. We are not charity.
D Consequentialist argument *for* fee reduction—Dr. D. believes rejecting patients' request to make a reduction would damage our reputation and would be harmful for our profession in long-term.

Respondents were asked to rate these four approaches according to their own opinion and intuition in such a way that the first rank is the most favorable option for them.

Data was entered and analyzed in SPSS 16.0. Statistical analysis of the ordinal data was done using non-parametric tests including Friedman and Mann–Whitney U tests.

## Result

The questionnaire was filled out by 157 respondents, including 121 sixth-year dental students and thirty-six tutors of Mashhad Dental School. Ninety-four respondents (59.9%) were female.

**Fig. 1:**
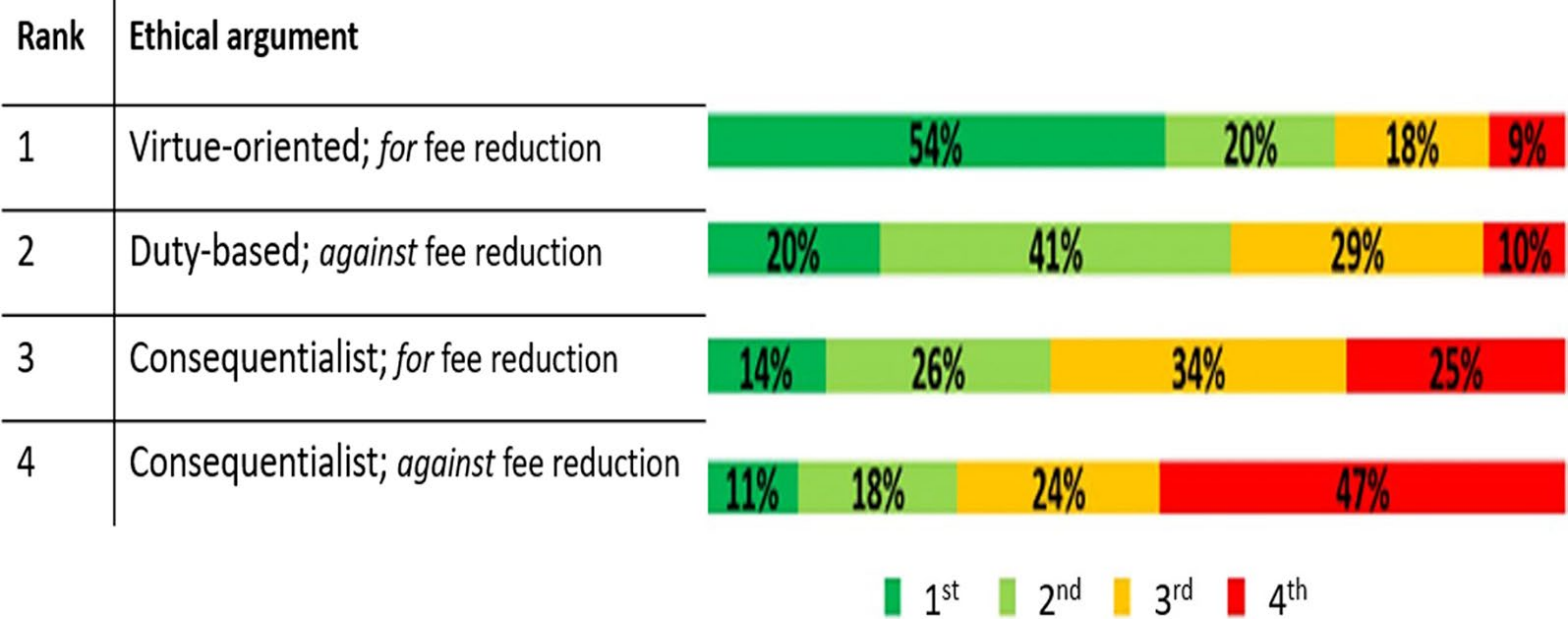
Descriptive ranking of the suggested approaches

The second option, which accounted for the virtue-oriented argument for fee reduction, was chosen as the first best option by 54 percent of respondents. The option D, accounted for the consequentialist argument against fee reduction, was selected as the least favored option by 47 percent of respondents. Figure 1 shows the descriptive report of proportion of rankings for each option.

The difference between rankings of four options was statistically significant (*p* value < 0.001*)*. The most favorable option, i.e. virtue-oriented argument for fee reduction, had the mean rank of 1.85. It followed sequentially by duty-based argument against, consequentialist argument for, and consequentialist argument against fee reduction. Table 1 displays the mean rank of four options.

**Table 1:** Mean ranks of the options of the vignette

Order	Option	Mean Rank
1	B—virtue-oriented argument for fee reduction	1.85
2	C—duty-based argument against fee reduction	2.32
3	D—consequentialist argument for fee reduction	2.74
4	A—consequentialist argument against fee reduction	3.09

No statistically significant difference exists neither between male and female respondents (*p* value = 0.60), nor students and tutors (*p* value = 0.22).

## Discussion

This study examined the ethical response and moral justification of dental students and faculty members when confronted with controversial issue in dentistry—fee reduction. It revealed that the majority of dental students and tutors in the second biggest city of Iran believe that accepting patients’ request for a fee reduction is the ethically preferable behavior. Almost 68 percent of participants selected either a virtue-oriented (54%) or a consequentialist (14%) argument for fee reduction as their best option.

Virtues define how we behave when no one else is watching, and generally are described as the qualities or excellences of character to which humans ought to aspire. As described by Aristotle, and extrapolated by medieval philosophers [13], it refers to a trait or character that is deemed to be morally good and thus is valued as a foundation of a principled life. Virtue considerations include reflection on how each act shapes the moral agent. In our survey a virtue ethics approach was the most favored one among the options offered. The consequentialist approach judges an action based on its consequences. This approach scored lowest among the options. Seventy-two percent of respondents regarded the consequentialist arguments, either for or against fee reduction, as the least justifiable response. There was no significant difference, neither between male and female respondents, nor between faculty members and students. We suggest that this may be a sign that there is convergent ethical intuition of the participants.

Similar arguments about other ethical and professional dilemmas arise in previous studies. For example, the same consequentialist approach at option D seems to be taken by Ayn, et al., who puts emphasis on improving communication skills in dentistry education [7], or by Gosden, et al., stating that clinical behavior of patients is affected by the method of payment [14]. The other consequentialist argument, put forward in the option A, is also in line with some suggested cost control strategies [15].

Yet the majority of our students and tutors acted in accordance with virtue, rejecting consequentialist arguments. The virtue-oriented approach has also been the mainstream attitude of many medical ethicists and is taken by many who discussed ethical aspects of dental practice [8, 16, 17]. However, it could be reasonably argued that there is a constant interplay between virtue, moral duty and consequentialist doctrine during fee reduction. Further investigation could be pursued to establish criteria for determining which patients will receive free or reduced-fee care. It would help individual dentists contribute in helping to overcome barriers in access to dental care.

The main limitation of our study was related to using the plain short vignette, which for the sake of encouraging compliance, did not involve the complex factors interplaying in the real clinical settings. There are a bunch of different patient- and dentist-factors that could affect the communication between patient and dentist during fee reduction. Issues such as the alternative treatment planning by dentists, bargaining attitude of patients, and the extent of the fee reduction could be incorporated in developing the vignettes in next studies. Considering such complexities would be helpful for simulating real life scenarios and making participants careful ethical deliberation.

Another limitation of our study was the potential difference in training about ethical schools of thought for different populations of students. There was also no comparison between senior and junior students.

Creating imaginative ways of thinking about common, yet important dilemmas, such as fee reduction is important. More study will need to be done to explore whether reducing the fee for dental work will reduce the value of the work among patients; whether asking for a fee reduction will become a habit; whether dentists will begin to expect this from their patients, and how any these questions, by focusing on the materialistic view of the profession of dentistry diminishes its nobility as a calling.

## Conclusion

We argue that using this case study provides a basis for useful discussions in ethics courses for dental students. It is also suggested that such financial issues be considered as a part of the professionalization and ethical training within the dental student's curriculum.

## Supplementary information

**Additional file 1**. The vignette used as the questionnaire of the study.

## Data Availability

The dataset supporting the results of this article are available from the corresponding author on reasonable request.

## Abbreviation

IRB: Institutional Review Board
