# Supplementary material for: Just say “no”: Can dentists refuse care on the basis of finances? A survey using an ethical vignette in an Iranian Dental School
Source: BMC Med Ethics. 2020 Oct 31;21:109. doi: 10.1186/s12910-020-00554-7 (PMC7603726; doi:10.1186/s12910-020-00554-7)
Supplement: Supplementary file 1 — Additional file 1. The vignette used as the questionnaire of the study. [file 12910_2020_554_MOESM1_ESM.docx]

***The vignette***

*It was a cold winter night. Five friend who were all dentists got together and were talk to each other.*

**Dr. E.:** Believe me… if not once a day, at least once in every two or three days, a patient at my office asks me for a fee reduction. “I couldn’t afford… my wife is ill… my husband is unemployed… it’s too expensive for me… so on and so forth.” I frequently listen to words like this! And you know, it’s a real dilemma for me. I still don’t know how to handle it. To accept their request or not?

**Dr. A.:** It has been the case at my office too. And I made my decision.
“Impossible!” It was what I told my assistant to say to such patients always. We have fixed tariff for each treatment, not because I am a mean person or too concerned about my earning… The reason is that if I make reduction, it might become a routine for patients to expect me and other dentists to reduce the fee. And after a while, at a broader level, such expectations might result in presenting high prices at first encounter which would be a bit modified after reduction. I don’t like such liar market.

**Dr B.:** You may be right. But when I see a respectable person, either rich or poor, swallows his or her pride and make a request for fee reduction, I feel that the humane behavior is to be sensitive to his or her request. Anyway, I think moral conscience dictates what to do in such a situation. I am sure that an honest and sensitive dentist would make a wise decision.

**Dr. C.:** You know Dr. B… what you do is much higher than what we are obliged. Here would be a paradise if every dentist behaves like you. But real life is something else. Our duty is to act in line with our professional obligations. We are not wanted to do what government or charities should do. My duty is to provide high-quality standard treatment for my patients and not charging them more than a fair fee. But it‘s not necessary at all to make fee reduction.

**Dr. D.:** Interesting arguments! Anyway, in my eyes, not making reduction in response to such a request, might break the trust and respect between patients and their dentists. It boomerangs on us.

...

*The discussion went on. The friends had tea together and defend their position.*

**Rank the four arguments made by Drs A, B, C, and D according to the level of your agreement with their arguments.**
